# A mixed-methods evaluation of the quantitative and perceived impact of Brexit on UK oncology clinical trial initiation between 2011 and 2020

**DOI:** 10.1080/20523211.2026.2673689

**Published:** 2026-06-15

**Authors:** Meena Arora, Sean Elliott, Adrian Kilcoyne, John Bolodeoku, Assem el Baghdady

**Affiliations:** a Centre for Pharmaceutical Medicine Research, Institute of Pharmaceutical Science, King’s College London, London, UK; b Fortrea Clinical Research Ltd, Leeds, UK; c Cellectis Inc., New York, NY, USA

**Keywords:** Brexit, clinical trials, oncology, clinical trial volume, Horizon

## Abstract

**Introduction:**

This study explored the perceived impact of Brexit on oncology clinical trial initiation in the UK, with comparative analysis against Spain and France, and evaluated potential impact on the UK's leadership position in clinical research. A mixed-methods approach was employed comprising: (1) analysis of oncology clinical trial registrations (2011–2020) using ClinicalTrials.gov for the UK, Spain, and France; (2) a stakeholder survey on Brexit-related impacts (*n* = 22); and (3) a systematic literature review (SLR) conducted in line with PRISMA guidelines using PubMed, Web of Science, Google Scholar, and Embase (2011–2021), with critical appraisal via CASP criteria.

**Discussion and Analysis:**

Clinical trial volume in the UK declined from 2017, returning to 2011 levels; a trend not mirrored in Spain and only transiently observed in France. From survey results (*n* = 22), stakeholders acknowledged a negative impact on EU collaboration, highlighting the importance of Horizon Europe programme funding and the role of European Research Networks (ERNs). The SLR identified concerns about increased trial costs, regulatory burden, and reduced patient access to innovative treatments in the UK post-Brexit. Alignment between UK and EU clinical trial regulation was widely seen as essential. UK trial activity shows a marked post-Brexit decline compared to France and Spain. Stakeholder and literature data suggest this trend may affect the UK's access to clinical trials for patients. The negative perception of Brexit's impact on collaboration, funding, and regulatory coherence warrants further investigation and policy response.

**Conclusion:**

The decline in UK clinical trial volume to pre-2011 levels poses a risk to patient access and the UK's status as a global leader in drug development. Sustained collaboration with the EU is viewed as vital for mitigating these effects and maintaining research competitiveness.

## Introduction

It is indisputable that clinical trials provide benefits to both patients and clinicians. For patients, clinical trials provide early access to innovative medications, which in fields such as oncology remain an area of high unmet medical need. For clinicians, clinical trials provide access to innovative medicines, professional development opportunities, build research capabilities and publications, as well as access to vital streams of revenue for institutions. Cancer clinical trials are imperative, particularly in rare and childhood cancers which have eluded treatment for decades. This is a confirmed area of high unmet need that was recognised by the UK Government in the recent UK Commons Cancer Services report from April 2022. The report stated:

Despite progress, UK patients still have much worse five-year survival rates for many cancers than those in similar nations. Early diagnosis and fast and equal access to the latest treatments for all patients is key to reversing poor trends in National Health Service (NHS) cancer care. (House of Commons, 2022)

A report in 2021 from the Association of the British Pharmaceutical Industry (ABPI) (ABPI, 2021) demonstrated that the number of clinical trials initiated in the UK declined from a figure of 667 in 2017 to only 508 in 2020 – a decline of 24%. If COVID-19 studies are excluded, then the total initiated in 2020 was 440 trials – a decline of 34% in just three years, with a steeper decline if only oncology trials are considered (ABPI, 2021).

The short-term impacts of Brexit have been evaluated in a range of studies and reports, including a recent study evaluating oncology clinical trials across the EU (Arora et al., 2024). Brexit initially created significant disruptions in UK oncology research. VanHelene et al. (VanHelene et al., 2024) demonstrated that the UK experienced a notable decrease in Phase 3 clinical trial initiations immediately post-Brexit in 2020, followed by a rebound in 2021 driven predominantly by industry-funded trials. The O'Shaughnessy review (Department of Health & Social Care, HM Government, 2023) revealed a 44% drop in patients enrolled in commercially led studies supported by the National Institute for Health and Care Research (NIHR) between 2017–2018 and 2021–2022.

Given these observations, following Brexit, it is crucial to understand the impact on the UK in terms of being able to retain world-class leadership in oncology research with the consequent benefits for patients’ ability to access innovative therapeutics. Cancer trials sit at the intersection of high public health burden, economic importance, rapid innovation, and heavy regulatory dependence, so any disruption or opportunity from Brexit is likely to be especially visible in this therapy area. (VanHelene et al., 2024) Similarly, cancer is a leading cause of morbidity and mortality in the UK, with large direct NHS costs and major productivity losses, so disruption to oncology research has disproportionate health and economic consequences compared with many other therapy areas, suggesting that exploring specific impacts on oncology research is a rational approach (University of Leeds, n.d.).

The purpose of the study was twofold. Firstly, to determine whether Brexit has any significant impact on the volume of cancer research conducted in the UK, following the UK's vote to leave the European Union (EU) in 2016, as compared with the annual number of oncology clinical trials that took place in the UK in the five years prior to the EU referendum. Secondly, using a survey of key stakeholders, perceptions of the impact of Brexit were explored against a systematic literature review of published perceptions and concerns to help elucidate further insights.

Other objectives of this research were to:

- Explore the impact of the decision and implementation of Brexit on the UK's collaborative relationship with other countries in facilitating oncology clinical trials.
- Investigate how legislation introduced by the Medicines and Healthcare Products Regulatory Agency (MHRA) in 2022, decoupled from the European Medicines Agency (EMA) post-Brexit, has incentivised cancer research in the UK or not.
- Identify the main challenges to oncology stakeholders because of Brexit and explore how these challenges can be addressed through legislation and policy.

## Methods

The research programme was divided into three parts: review of (1) clinical trial databases; (2) stakeholder survey and (3) systematic literature review.

### Clinical trial database review

Clinical trial initiation and status were evaluated using www.ClinicalTrials.gov as a comprehensive repository of ongoing and completed clinical trials. ClinicalTrials.gov is the largest registry of private and public funded clinical trials (ICF Inc., 2023), which is fully accessible and contains all clinical trial phases, unlike European equivalents EudraCT and EU Clinical Trials Regulation (EU CTR). Therefore ClinicalTrials.gov was used as the single source of data as it captures all clinical trials and avoids the potential of double counting if other sources were also used. Interventional studies with patients recruited in the UK were chosen for evaluation from Phase 1 to Phase 4.

The comparator countries Spain and France were chosen as countries that are broadly comparable to the UK in terms of population, clinical trial initiation and completion, and cancer incidence rates. Both Spain and France have healthcare systems that are predominantly publicly funded, which is an important factor in oncology research as this provides a comparable pool of potential patients. Other published research corroborates comparator choice countries with the macro-developmental analysis of European countries in haemato-oncology research (Arora et al., 2024).

With the referendum to leave the EU completed in 2016, a timeframe for clinical trial evaluation from 2011 to 2020 was chosen as appropriate to gauge a baseline pre-Brexit, the interim period while Brexit was being implemented, and in the immediate post-Brexit period as final agreements in 2020 were secured. Cancer trials that were suspended, terminated or withdrawn were also included to most accurately reflect the total number of oncology studies that have started in the UK each year since 2011.

We determined a search strategy that included both free-text words and medical subject headings (MeSH). The search criteria used to interrogate ClinicalTrials.gov in a search performed on 25th July 2021 included: ([‘cancer' OR ‘tumour' OR ‘tumor' OR ‘oncology’) AND ‘(interventional studies [clinical trials]') AND (‘studies with results' OR ‘studies without results') AND (‘not yet recruiting' OR ‘recruiting' OR ‘enrolling by invitation' OR ‘active, not recruiting' OR ‘suspended' OR ‘terminated’ OR ‘completed' OR ‘withdrawn') AND (‘United Kingdom') AND (‘Phase I' OR ‘Phase II' OR ‘Phase III' OR ‘Phase IV') AND (‘industry’ OR ‘all others [individuals, universities, organizations]'). Studies were screened for duplicates and a PICOS framework applied (see Supplemental Material Table S1).

### Survey of perceived impact on oncology clinical trials

A bespoke survey of 10 questions was developed specific to this research. As this was a one-off piece of research, validation of the questionnaire was not considered necessary. The questionnaire was emailed to identified key stakeholders in the oncology community including the MHRA, cancer trial sponsors, academic organisations, Cancer Research UK and patient advocacy groups. The survey questionnaire was devised on a Likert scale, e.g. very confident to very uncertain, conducted in English, was not anonymised, and can be found in Supplemental Material. The survey explored the areas of:

- Perceived impact of Brexit on the UK's position as a primary European player in cancer research (EduRank, 2023)
- The potential for future collaboration with the EU post-Brexit
- The potential for the UK to seek closer collaborative ties with non-EU countries
- Confidence of stakeholders that funding shortfalls caused by Brexit can be met by the UK Government

The survey was intentionally designed with limited multiple-choice questions to increase the likelihood of completion and return by identified stakeholders. Responses were scored according to a 5-point Likert scale so that responses could be evaluated through frequency analysis. Minimal risk ethics approval was sought from the King's College London Research Ethics Committee and granted on 8 July 2021.

### Systematic literature research

#### Protocol

The systematic literature review was conducted in line with the Preferred Reporting Items for Systematic review and Meta-Analyses (PRISMA) guidelines (Moher et al., 2015). Institutional Review Board (IRB) approval was not required.

#### Search strategy

Searches were conducted using PubMed, Web of Science, Google Scholar and Embase. All authors determined a search strategy that included both free text words and medical subject headings (MeSH). Literature searches were conducted between the 8th and 10th of August 2021, and focused solely on peer-reviewed primary publications. Search terms used were: ([‘Brexit'] AND/OR [‘European Union' OR ‘EU'] AND [‘cancer' OR ‘tumour' OR ‘oncology'] AND [‘clinical research' OR ‘clinical trial*' OR ‘clinical study' OR ‘cancer research'] AND [‘United Kingdom' OR ‘UK']). Whilst it was noted that the term Brexit was not in common parlance until 2016, the search parameters were applied to capture both literature referring to oncology clinical trials and that relating to the specific impact of the Brexit referendum on oncology trial initiations.

#### Publication selection

Inclusion criteria for publications are shown in the Supplemental Material Table S2. Search parameters were limited to material published in English between 2011 and 2021. First pass evaluation was undertaken by an independent reviewer followed by a second pass evaluation of published full texts to determine their eligibility and the strength of the evidence. Any disagreements were resolved through author discussion.

All publications meeting the eligibility criteria were reviewed against the Critical Appraisal Skills Programme (CASP) Systematic Review checklist by one reviewer to evaluate the quality of each publication (CASP, 2023). Only publications scoring a minimum quality score of 5 were included for full review. Extracted publications are shown in Supplemental Material Table S3. A narrative synthesis approach was used to analyse retrieved publications (Popay et al., 2006). The review satisfied the criteria of the Risk Of Bias In Systematic reviews (ROBIS) tool (Whiting et al., 2016). Abstracts of all literature sources returned were reviewed to remove duplicates and evaluated against specific inclusion criteria.

## Results

### Evaluation of clinical trials being conducted in the UK

**Figure 1.**
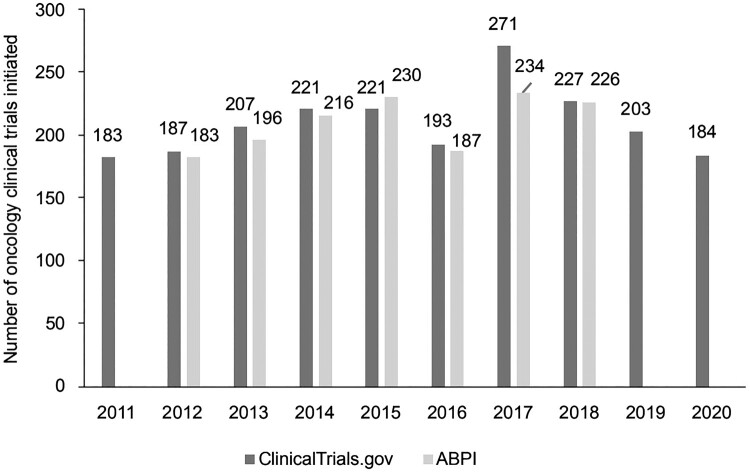
Number of oncology clinical trials initiated in the UK between 2011 and 2020 according to ClinicalTrials.gov compared with those from the ABPI between 2012 and 2018.

Evaluation of ClinicalTrials.gov established the overall pattern of oncology clinical trials from 2011 to 2020 (Figure 1). From 2011 to 2015, a steady increase in oncology trials was observed. However, in 2016, the year of the Brexit referendum, a 13% decline was seen in oncology trial initiations in the UK when compared with 2015. A transient rebound in clinical trial numbers in 2017 was followed by a consistent downward trend to 2020, with only 184 new cancer studies initiated in 2020, in contrast to the peak figure of 271 new oncology trials recorded in 2017. These data were comparable to those observed from the Association of British Pharmaceutical Industry (ABPI) report published in 2020 (ABPI, 2020). However, the decline in cancer study numbers in 2016 in the report at almost 20% (221 new oncology trials in 2015 as opposed to 193 in 2016) was higher than the 13% reduction indicated by the ClinicalTrials.gov data, with a smaller rebound increase in 2017 (Figure 1). In the report from the ABPI in 2024 (ABPI, 2024), it was noted that the number of clinical trials initiated had fallen across all therapy areas from 2017 and only started to recover thereafter, with very recent data from ABPI indicating a continued trend in improvement (ABPI, 2025).

**Figure 2.**
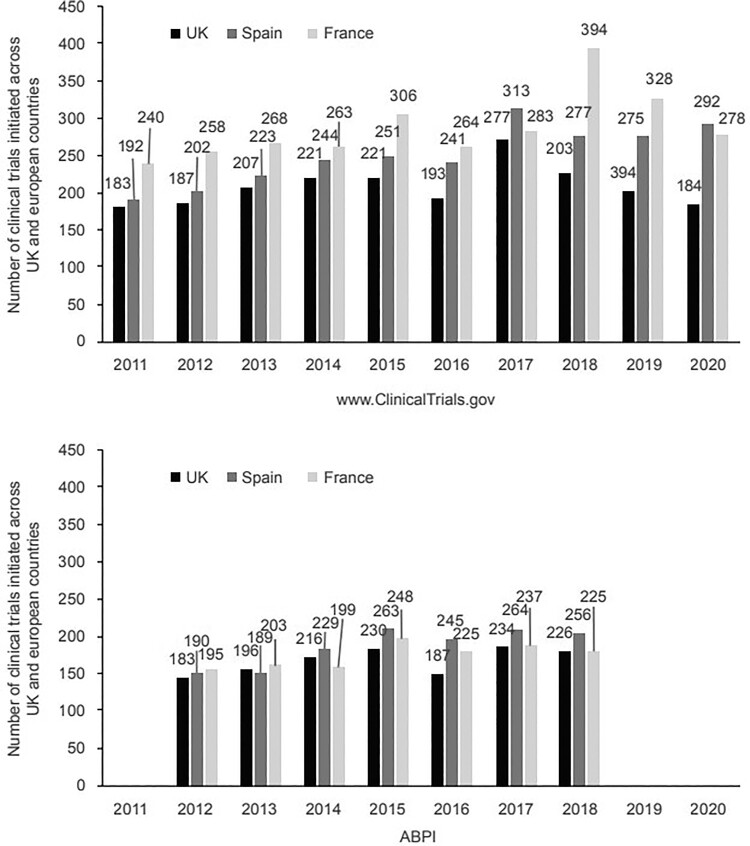
Number of oncology clinical trials initiated in the UK, Spain and France between 2011 and 2020 according to ClinicalTrials.gov compared with those from the ABPI between 2012 and 2018.

Comparisons of UK performance against reference countries Spain and France were undertaken (Figure 2).

In the UK, it is apparent that gains in clinical trials between 2011 and 2015 were lost by 2020, where clinical trial initiations were comparable to 2011, reflecting a sharp decline of 32% from 2017. Whilst some level of decline was observed in France in 2016, it was not the level observed in the UK, and it appeared to be recovering. The number of clinical trials was only 9% lower than that seen in 2015, suggesting this trend towards recovery of clinical trial implementation. The numbers of early phase clinical trials in France were lower than in other EU countries (d’Andon et al., 2021), which may reflect the fallout from the first-in-human (FIH) clinical trial tragedy in 2016, which led to changes both in France and at the European Medicines Agency for how these studies should be conducted (Inspection Générale des Affaires Sociales, 2016; van der Laan et al., 2020; van Gerven & Bonelli, 2018). By contrast, in Spain, the number of oncology clinical trials was relatively stable over the period of study, with a slight upward trajectory over time (2011 = 183 trials; 2017 = 313 trials, and 2020 = 292 trials) (Figure 2). These data collate together oncology clinical trials from all phases, including those safety trials in healthy volunteers and early efficacy trials in oncology in phases 1 and 2, where trials may be easier to implement, requiring smaller patient numbers. It could be suggested that the decline observed here cannot be attributed only to Phase 3 trials in the UK, and may be a reflection of an overall trend in clinical trial initiation. However, previous data by our group (Arora et al., 2024) indicated that Brexit impacted Phase 3 studies in the UK disproportionately. In this previous study where data were collated over a similar timeframe, it was noted that the attractiveness of the UK as a clinical trial destination was under threat due to the impact of bureaucratic barriers compounded by the threat of Brexit.

### Survey of perceived impact on oncology clinical trials

**Figure 3.**
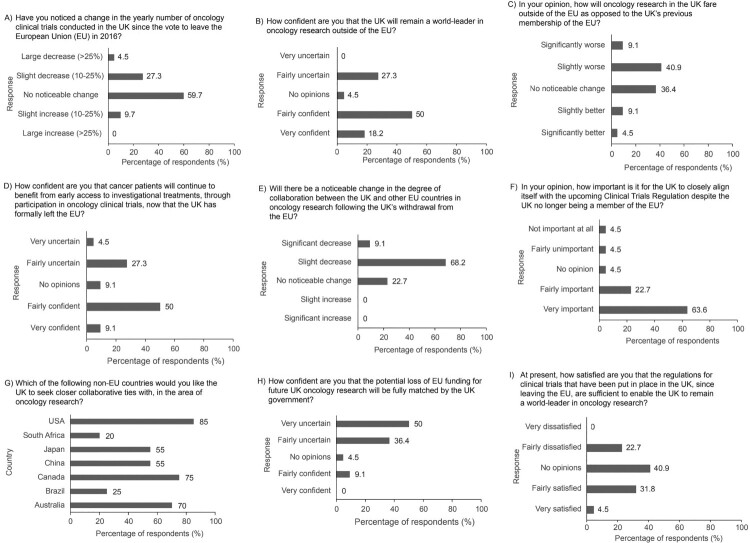
Responses to survey questions (*n* = 22).

Influence mapping of stakeholders participating in the survey allowed for an understanding of their potential to initiate legislative changes in the UK and the degree to which their work is focused solely on cancer research (details of stakeholders and stakeholder mapping are shown in Supplemental Material Fig. S1). Of the 80 surveys distributed, 22 were completed and returned, representing a response rate of 27%. Respondents included clinicians (oncologists working within NHS and private facilities) (40.9%), pharmaceutical companies (27.3%), clinical research organisations (CRO) (13.6%), academic institutions (13.6%) and cancer charities (4.5%). The MHRA was one of the institutions sent the questionnaire but did not respond. The 27% response rate is consistent with typical rates for research surveys (25–29%). Responses to survey questions are shown in Figure 3A–I.

From the scoring of the survey responses, there was a confidence among 59% of respondents that, following the Brexit referendum, there was no appreciable change to oncology clinical trials; although a quarter (27%) noted a slight decline in new studies starting after 2016. There was a sense of optimism among respondents that the UK would remain a world leader in oncology trials, with 50% fairly confident of this and 18% very confident. A third of respondents (36%) felt Brexit would have no effect on UK oncology research, but collectively, 50% felt it would be slightly or significantly worse post-Brexit, with only 13% believing Brexit would improve UK research. At the time of this survey, half of respondents were confident that the UK would continue to gain early access to medical innovation, with only a quarter fairly uncertain (27%). Consistently, respondents agreed that Brexit would decrease the degree of UK-EU collaboration, with more than two-thirds (68%) estimating this to be slight. A similar proportion agreed that the EU CTR, enacted in 2022, would be very important to the UK with close alignment essential.

Whilst elaborate statistical analyses are not possible on a dataset of this size, and individual question data were not collated by participant occupations, it is noted that 68.2% of all respondents were clinicians (40.9%) or involved in clinical trials through the pharmaceutical industry (27.3%) and a further 13.6% were derived from CROs. Given this, it can be inferred that results are broadly representative of individuals with intimate knowledge and insight into the oncology clinical trial landscape in the UK, although this cannot be known definitively. Further study would be needed, using a large cohort, drawn from a boarder demographic with correlation of perception to individual occupation categories to be sure of any trends.

### Systematic literature search

**Figure 4.**
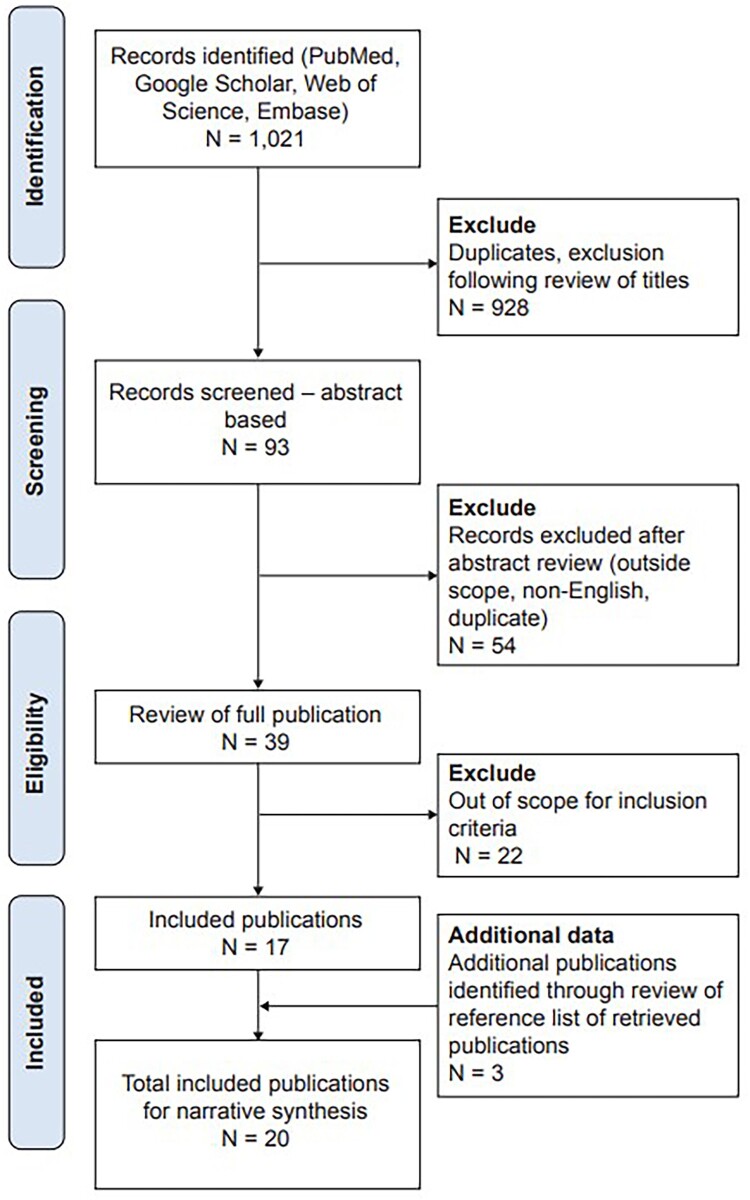
PRISMA flowchart depicting the selection of studies and data in the analysis.

Searches returned an initial total of 1021 publications, of which 941 were indexed on Google Scholar, 57 on PubMed Central, 12 on Embase and 11 papers were retrieved from Web of Science. After assessing the eligibility of these papers through the use of the inclusion criteria, 17 were included in the narrative synthesis. An additional 3 papers, identified by searching the reference lists of the 17 retrieved articles, met the inclusion criteria leading to the final selection of 20 studies (Figure 4)*.* An overview of included studies and their CASP scores is shown in the Supplemental Material Table S3.

All papers finally chosen for inclusion achieved a minimum score of 5 using the CASP Systematic Review Checklist (mean CASP score 8 and median score of 8.5) (Breckenridge & Feldschreiber, 2019; Hatswell, 2017; Hawkes et al., 2018; Hervey et al., 2021; Lawler et al., 2018; Matthews & Williams, 2018; Mimoglu et al., 2017; Reijntjes et al., 2018; School of International Futures, 2021; Selby et al., 2016; Tsagakis & Papatriantafyllou, 2020; Vousden, 2019). Three papers achieved the maximum CASP score of 10, with a further 9 attaining a score of 9. Some publications merited a score of 0 for individual CASP criteria as relevant information needed for a score was not provided. Eight publications (40%) addressed the significant uncertainty that Brexit posed in relation to the conduct of future clinical trials in the UK.

The SLR provided insight into the collaborative partnership between the UK and the EU pre-Brexit, the potential for revenue loss to the UK following Brexit or increased costs following Brexit and the potential for increased bureaucracy with respect to medicine approvals and patients’ access to them following the vote to leave the EU (Breckenridge & Feldschreiber, 2019).

From 2004 to 2016, more than 4800 clinical trials were pan-European involving at least 2 EU countries and benefiting patients across Europe, including the UK. Collaboration across European countries was most notable in rare or childhood cancer, where patient populations for unilateral studies would have been insufficient (Lawler et al., 2018; Varnai et al., 2017).

The loss of revenue to the UK for cancer research as a consequence of Brexit was evaluated in six publications. As the fourth biggest beneficiary of EU research funding (£6.5 billion from 2007 to 2013) overall, with up to 40% of this invested directly in cancer research, experts published concerns and warnings of the direct impact to patients of the loss of EU funding (Lawler et al., 2018; Mimoglu et al., 2017; Selby et al., 2016). A need to negotiate for either continued access to EU funding or for the UK Government to mitigate the shortfall was stridently raised (Lawler et al., 2018; Mimoglu et al., 2017; Selby et al., 2016; Tsagakis & Papatriantafyllou, 2020; Tumiene et al., 2021; Vousden, 2019).

The desire of Brexit to ‘take back control’, (HM Government, 2018) particularly in the form of legislation, caused concern for patient access to medications. The lack of clarity around the UK aligning with the EU Clinical Trial Regulation (CTR) caused notable apprehension (Burki, 2018), particularly as divergence from the EU CTR may lead to significant delays in patient access to innovative therapies (Breckenridge & Feldschreiber, 2019; Buckland, 2017; European Observatory on Health Systems and Policies, 2019; Fahy et al., 2021; SOIF, 2021). These delays were estimated to be in the range of at least a year and perhaps two (Breckenridge & Feldschreiber, 2019; Buckland, 2017; European Observatory on Health Systems and Policies, 2019; Fahy et al., 2021; SOIF, 2021). It is believed that such delays will weaken the UK's global position with respect to clinical trials and access to new medical treatments.

It is suggested that additional UK bureaucracy and regulatory requirements will further hinder access to medicines and increase healthcare costs. These costs need to be met solely by the NHS if medicines are sanctioned for use by the National Institute for Health and Care Excellence (NICE) (Hatswell, 2017). With direct increased healthcare costs also come reduced levels of discount from parallel importing of medicines. It was suggested that the UK's departure from the EU would have a detrimental effect on parallel importing. Parallel imports lower the costs of medicines to the NHS, providing estimated savings from 2004 to 2009 of £880 million (Lorgelly, 2018). To date, no changes to current exhaustion rights legislation have been made, but it is unclear how long this will continue (Breckenridge & Feldschreiber, 2019; Buckland, 2017; Burki, 2018; European Observatory on Health Systems and Policies, 2019; Fahy et al., 2021; SOIF, 2021).

Among the literature evaluated there is limited to no delineation of impact of Brexit by phasing of clinical trials, with most literature exploring clinical trials overall. However, previous data we have published suggests that Phase 3 trials were disproportionately affected over the timeframe reported, with Phase 1 largely unaffected by political decisions and Phase 2 studies impacted to a lesser degree than Phase 3 (Arora et al., 2024). These data are supported by very recent data from the ABPI (ABPI, 2025) which demonstrated a broadly stable implementation in all Phase 1 trials from 2012 to 2024 (not just oncology), but a decline in Phase 3 trials from 2018 to 2021 of 52% (2017 = 292 Phase 3 trials, 2021 = 140 Phase 3 trials) and Phase 2 trials of 34% (2018 = 268 Phase 2 trials, 2021 = 162 Phase 2 clinical trials. However, since 2021 rates of Phase 2 and 3 trials have improved although numbers of trials are not yet at 2018 levels.

## Discussion and analysis

Clinical trials require extensive investment in terms of time, resources and finance, which is outweighed by the potential benefits both to patients and society. Understanding the choice of specific countries to participate in clinical trials requires an understanding of the position of sponsors of studies in placement of global research, an analysis of the willingness of countries to invest in clinical studies, their landscape of current healthcare funding, clinical trial capacity from the perspective of the potential patient population and the attitude of healthcare professionals and the willingness of patients to be involved in research.

The UK is well placed for clinical trial implementation by merit of the National Health Service (NHS), whose infrastructure can provide a well-characterised patient population for clinical studies, combined with world class medical capability and patient care. These data suggest, even removing COVID-19 as a confounder, that the UK has lost some ground in terms of being a leader in oncology clinical trials over the last few years. Our data indicate that the timepoint for the UK's declining share of voice in launching new oncology clinical trials is 2016, coinciding with the referendum decision for the UK to leave the EU.

Up to 2015, there was a steady increase in the number of oncology clinical trials initiated in the UK, these data are in line with the UK being ranked as one of the top five countries globally for oncology research up to 2015 (ABPI, 2019). The sharp reduction in 2016 in the number of clinical trials initiated in the UK could be attributed to the unexpected outcome of the EU referendum result in June 2016, which resulted in a deep sense of uncertainty around complex issues such as drug importation, licensing and approval, as the potential loss of future EU funding and the possible divergence of the UK from the EU clinical research framework (Abbott et al., 2016). The number of oncology trials in the UK commencing in 2020 of 184 was comparable to the 183 initiated in 2011. Unsurprisingly, the number of clinical trials was reflected in the lower number of patients able to access oncology clinical trials, thereby limiting patient access to innovative therapeutic options for their cancer treatments. Our data are comparable with those outlined in the recent Nuffield report (Nuffield Trust, 2024 which demonstrated the UK experienced an immediate decline in phase III oncology trials from 2019 to 2020, followed by a strong rebound in 2021 to 89 industry-funded trials, the highest number recorded. However, this recovery masks concerning trends: non-industry academic trials declined from 10 (2019) to just 5 (2022), suggesting Brexit disproportionately affected research infrastructure beyond commercial interests. The UK's 90% reliance on industry-funded cancer trials versus 57% globally indicated vulnerability to commercial decisions. While global trends influenced these patterns, Brexit created unique regulatory uncertainties that deterred trial sponsors (Nuffield Trust, 2024). The data in the Nuffield report explored only industry-sponsored clinical trials, an important yardstick often of confidence as suggested by the authors, but the data presented in this study provide insight into oncology clinical trials including government, charity and industry-sponsored and suggest comparable trends.

We acknowledge that the decline in UK oncology trial initiations does not, in isolation, confirm a loss of global research leadership. Trial volume alone does not capture other critical dimensions such as trial quality, phase distribution, scientific output, or innovation in early-phase studies. Due to dataset limitations, we were unable to disaggregate by these broader indicators. Recent published research (Arora et al., 2024) offers a broader perspective on the evolving nature of UK research leadership. In addition, potential confounding factors including NHS budgetary pressures and delays associated with the Integrated Research Application System (IRAS) may also have influenced the observed trends.

The final data obtained in this study was in 2020, and it would be interesting to note if, following the end of the COVID-19 pandemic, oncology clinical trials remain at the levels of 2011. Clearly, the cut-off datapoint of 2020 is a significant limitation, and further analysis to explore trends in oncology trials beyond this point would be interesting – particularly the impact of COVID-19 research, which eclipsed other clinical trials for several years and only now has receded sufficiently in the background of overall clinical trials to explore specific trends. Future analyses in this regard would provide an insight into continued decline or if a rebound in oncology trial numbers is observed. A recent UK report from the ABPI (ABPI, 2024) suggests that clinical trial initiation is improving with 411 trials initiated in the UK in 2022 rising to 426 in 2023, data that are further supported by the ABPI's 2025 report (ABPI, 2025). However, the impact of this rebound on oncology trials alone is not demarcated, remains currently unknown, and would be interesting to explore in future studies. An interesting observation from this study is the uplift in clinical trial initiations in both France and Spain in 2017 and 2018. It is interesting to surmise that the uplift may be benefiting in part from the transition of trials from the UK; in part, be due to the Brexit referendum, as industry sought to protect interests by moving trial sites away from the UK until the post-referendum landscape became clear. However, this cannot be proven from our datasets, and in both countries, clinical trial initiations have plateaued to a stable state as seen before 2017 and 2018. We would be curious to note if clinical trial numbers have continued to grow for France and Spain after the UK left the EU or if the post-Brexit uplift was a temporary exception.

It must be remembered that the Brexit referendum was legislated in December 2015, with the vote taking place in June 2016, suggesting that perhaps confidence in the UK was weakened even before the outcome of the vote (Walker, 2021). This may explain the decline in 2016 followed by a temporary increase in 2017, once it became clear that exit from the European Union would take more than 4 years. From 2017 onwards a worrying downward trajectory in the number of UK clinical trials is clear which was not mirrored in reference countries of Spain and France, suggesting a uniquely UK parameter is driving this change. The ongoing decline in clinical trials after 2017 may reflect the lack of attractiveness of the UK as a clinical trials hub for oncology research because of its exit from the EU. The reduction in UK patient numbers enrolling in clinical trials was 37% from 2015 to 2016 which equates, in real terms, to 33,000 fewer patients able to receive innovative care as part of a trial. If this decline in the number of UK cancer studies were to continue from 2021 onwards, it would be of concern for oncologists and patients alike.

Between 2014 and 2020, UK-based scientific researchers had access to £1.1 billion from Horizon 2020 alone, which was the EU's flagship research programme (Benjamens et al., 2020). More specifically, in 2018 UK cancer research received £40 million from the EU, as well as the option to participate in pan-EU oncology studies (Macmillan Cancer Support, 2018). The UK has benefited greatly from EU funding whilst being a member of the EU and a leader in clinical research, but since the decision to leave the EU, there has been great unease amongst UK oncologists regarding the source of future research funding (Milne-Ives et al., 2020).

Survey respondents echoed the sense of unease, with half stating they were unclear if funding shortfalls would be met by the UK Government. Announcements in 2017 that the UK would be an associate member of the Horizon scheme, allayed these fears (European Commission, 2021). Evaluation of Horizon 2020 indicated UK Cancer researchers reaped rewards of up to £500 million in funding (Kirby 2025) results that are substantiated by a very recent report from the UK Government that indicates the role of Horizon funding as a driver of academic and clinical impact of UK research but a residual instability continues to remain (HM Government, 2025). Interestingly, survey respondents seemed more optimistic about a post-Brexit landscape than reports and publications suggested; exploring this possible disconnect would be interesting for future studies. It was interesting that more than 60% of survey participants felt confident or very confident that UK citizens would still be able to effectively access participation in oncology trials. This may be linked to the finding that many felt clinical trial development and implementation would be unhindered by Brexit in the UK. However, this finding is at odds with those uncovered through the published literature.

The data presented here suggest that the combination of regulatory isolation, supply chain fragmentation, and declining academic research capacity extends the impact of Brexit beyond an immediate disruptive impact on clinical trials to a longer-term impact on patient access to treatment. These data are consistent with those presented by other authors (Department of Health & Social Care, HM Government, 2023; Lamberink et al., 2022; Nuffield Trust, 2024).

There are several limitations within this study. The survey was limited by the response rate of 27% (*n* = 22/80) and the limited number of respondents, therefore considered modest. In addition, the questionnaire was not anonymised and distributed by email only to identified stakeholders. No response was obtained from the MHRA and so the impact of their input cannot be gauged. It is possible that responses from the MHRA given the timing of the UK's exit from the EU, would have been inappropriate or sensitive (the survey was circulated in July 2021 onwards) although this can only be surmised rather than known. Consideration should also be given that this was a postgraduate single research project, whereby the survey was designed specifically for this purpose. Similarly, the survey questions did not delineate between company-sponsored trials and those with independent funding from government or charities; an analysis of this would be of interest.

Our systematic review includes studies published up to December 2020. For a systematic literature review, usually two or more reviewers would evaluate publications to minimise bias. The use of only one reviewer in this study may be considered a limitation and the risk of bias cannot be ruled out. Similarly, the survey was hindered by a small distribution and limited response rate, and by not having any input from regulators to explore all perspectives on the questions posed. Very few respondents were from the charitable sector (4.5%), and this reflects their issues with resourcing in answering questions and their reliance on UK government funding, which may discourage them from commenting on issues such as Brexit (Casassus, 2018; Luengo-Fernandez et al., 2015).

The UK has transitioned to using global trial registries including ClinicalTrials.gov, the ICTRP from the World Health Organization and the UK registry offshoot the ISRCTN. These data reflect clinical trials registered with ClinicalTrials.gov, which is relevant now that the UK does not have access to the EU clinical trial database EudraCT. Brexit has created unique challenges for UK clinical trials, particularly regarding EU transparency requirements that mandate registration in ICTRP-recognised databases for any trial data used in EU applications. ISRCTN, as the UK's primary registry, ensures clinical trials meet standard requirements and provides and maintains valuable international research connectivity and regulatory alignment despite EU separation. UK trials registered in ISRCTN remain visible to international researchers, sponsors, and patients through the ICTRP search portal, preventing Brexit from isolating UK research. Given the depth of the ISRCTN and ICTRP, it would be interesting in future studies to explore the impact of Brexit over a longer time span against France and Spain, but also against Europe more generally. In this way the body of evidence can be explored that complements both this study and that of VanHelene et al. (VanHelene et al., 2024)

Rare disease presents a particular challenge in all therapeutic areas of clinical research. Consequently, European Research Networks (ERNs) were established in 2017 connecting 900 research teams in 25 countries, with the UK being pivotal to their set up and leadership (Héon-Klin, 2017). Collaboration is essential in rare disease, with rare cancers forming up to 20% of all cancers and up to 30% of all cancer deaths (Blay et al., 2021). Only through collaboration can the needs of rare cancers be met. The most effective EU partnerships were apparent in rare and childhood cancers, where collaboration enables a full patient cohort to be established for study. From March 2019, however, NHS trusts were excluded from additional ERN funding in preparation for the UK leaving the EU, and UK involvement in ERNs was ended in early 2021 (Hawkes et al., 2018). The withdrawal of the UK from such important collaborative partnerships as ERNs raises concerns that fewer trials in rare disease will be conducted in the UK, the consequence of which is limited access to innovative therapies for British patients.

Researchers had considered that leaving the EU would increase costs of therapies as regulations and bureaucracy increase (Hatswell, 2017), and the UK Government's own modelling indicated this is likely to be true with an anticipated increase in medication costs of 5%, equivalent to an additional £400 million cost burden to the NHS. Costs to the NHS and the economy through leaving the EU have a direct impact on patients, and this is likely to continue. The European Clinical Trials Regulation (Clinical Trials Regulation EU 536/2014) was enacted into EU law in January 2022 and aims to provide a streamlined approach to clinical trials that make the EU an attractive trial location. At the same time, the UK launched a consultation led by the MHRA that was intended to make the UK similarly attractive to clinical trial partners. The Government response to this consultation was published in March 2023 (Medicines and Healthcare Products Regulatory Agency, 2023). As an immediate step the UK Government has pledged to support 5 headline commitments backed by funding of up to £121 million. This commitment revolves around the following:

- Ensuring patient safety is the focus of all clinical trials and that clinical trials benefit everyone
- Embedding public and patient involvement in clinical trials
- Creating a proportionate and flexible regulatory environment
- Cementing the UK as a destination for international trials
- Providing a framework that is streamlined, agile and responsive to innovation

Allied to these commitments is also a stated aim to stem the ‘brain drain’ and loss of scientists abroad as well as the obstacles confronting scientific talent migrating into the UK. Legislation to meet some of these key points is awaited, and while some guarantees were provided by Government in 2019, once legislation is announced it will be of interest to explore how aligned the UK framework is with that of the EU (Driskell et al., 2019). Any divergence from the EU CTR framework is concerning because separate clinical trial authorisations will need to be secured for the EU and the UK, increasing costs and administrative hurdles (Lorgelly, 2018; Shah & Mann, 2016). Further, divergence would likely mean availability of innovative therapeutics to EU patients first (∼500 million potential patients) rather than the UK (∼66 million potential patients). Early evidence suggests that the MHRA remains largely in step with the EMA (Hofer et al., 2022). The MHRA has implemented several mitigation strategies, including Project Orbis a programme to review and approve promising cancer drugs through regulatory collaboration outside of Europe, from January 2021, enabling concurrent oncology drug reviews with international partners (Department of Health & Social Care, HM Government., 2025; Hofer et al., 2022; Lythgoe et al., 2023a). Despite these mitigation strategies however, significant challenges persist. The Nuffield Trust (Nuffield Trust., 2024) reported that from December 2022 to December 2023, only four EU-approved drugs were authorised faster in Great Britain than elsewhere, while 56 were approved later and 8 did not receive approval at all. Another study (Lythgoe et al., 2023b) noted that UK medicine authorisations typically lag behind EU approvals, with the UK often relying on ‘reliance routes’ for faster approval of EU-authorised products. Similarly, the same group noted that European oncology approvals also lag behind those in the USA for both market authorisation and regulatory review (with the EMA being up to twice as long to review as the FDA; 426 days versus 277 days in one study [Lythgoe et al., 2022] and at least third longer in another study; 40 weeks versus 26 weeks [Vokinger et al., 2023]).

In the future, there are warnings of potential further isolation from EU initiatives like the Critical Medicines Alliance, which could prioritise EU countries during medicine shortages (Aggarwal et al., 2024). Whilst Project Orbis offers opportunities for maintaining competitiveness through international collaboration beyond the EU framework, a raft of potential future challenges remain, and it would be of interest to see how this initiative evolves over time (Department of Health & Social Care, HM Government, 2025).

To date, the UK has often been an early launch market for new medicines, as NICE assessments are highly regarded, and UK prices often serve as a reference point for other nations (Rémuzat et al., 2015). It has been suggested that unilateral regulatory marketing approvals in the UK will mean delays to treatment of 12–24 months (Hawkes et al., 2018). Limited ability to access studies or delayed access to medicines will be disastrous for oncology patients, especially those with rare cancers or children with cancer.

### Strengths and limitations of this study

- The strengths of this study are the use of robust databases that provide a reasonably accurate overview of clinical trial volumes in the UK and reference comparable countries like France and Spain.
- Limitations include the small distribution of the survey (*n* = 80) via email with no pre-survey publicity and lack of follow-up with respondents in person (either face to face or virtually).
- Other limitations include the lack of robust data exploring Brexit so that the systematic literature research was performed on publications on predicted opinion or modelling rather than Brexit outcomes.

It would be valuable to explore perceptions of the impact of Brexit in 2–5 years’ time when the full outcome of UK alignment to EU legislation, full and continued access to Horizon European funding and potential future collaboration with European Research Networks (ERNs) is established and functional.

## Conclusion

From this study, an observed decline in cancer clinical trials may suggest that the UK is lagging behind comparator countries when initiating oncology trials. The timeframe for this decline seemingly directly correlates in time with Brexit. The impact of this decline on UK revenue and the impact to patients’ access to innovative therapies currently remains uncertain. However, another study (Arora et al., 2024) has demonstrated that once oncology trials are initiated in the UK, completion is largely assured and the data provided is meaningful and relevant to further drug development and future patient care.

Our data suggest that there has been a lack of awareness of the impact of Brexit on the number of oncology clinical trials conducted in the UK, which could lead to complacency. Brexit was fully implemented more than four years ago, yet it is still unclear when the impact of the UK's decision to leave the EU will stabilise, particularly following the stopping of Section 6 commencement the Retained EU Law (Revocation and Reform) Act 2023 in September 2024. What is clear is that for stability to be achieved a closer collaboration with Europe will be required and the UK will need to have legislative alignment with Europe for UK patients to continue to receive early access to innovative medicines in a timely manner that is unhindered by additional bureaucracy. Such alignments to support closer collaboration between the EU and the UK would be regulatory enhancements, financial support mechanisms, MHRA-EMA collaboration, data/technology integration, and workforce/patient engagement. Implementation of these alignments will be of great interest to observe. Implementation of these alignments has shown some benefit recently with the increase in clinical trials in the UK with regard to the UK's global ranking in Phase II/III trials (ABPI, 2024).

## Supplementary Material

Supplemental Material

## Data sharing

All data are available upon reasonable request to the corresponding author.
