# Supplementary material for: A mixed-methods evaluation of the quantitative and perceived impact of Brexit on UK oncology clinical trial initiation between 2011 and 2020
Source: J Pharm Policy Pract. 2026 Jun 15;19(1):2673689. doi: 10.1080/20523211.2026.2673689 (PMC13270869; doi:10.1080/20523211.2026.2673689)
Supplement: Supplemental Material [file JPPP_A_2673689_SM1876.docx]

**Supplementary materials**

**A mixed‑methods evaluation of the quantitative and perceived impact of Brexit on UK oncology clinical trial initiation between 2011 and 2020**

**Arora M (0009-0007-7608-457X),^a^* Elliott S (0009-0008-3283-6087),^b^ Kilcoyne A (0009-0002-7882-8927),^c^ Bolodeoku J (**[0000-0003-4261-9285](about:blank)**),^d^ el Baghdady A (0009-0004-4673-5034)^e^**

^a^Meena Arora, Visiting Lecturer, Centre for Pharmaceutical Medicine Research, Institute of Pharmaceutical Science, King’s College London, UK; ^b^Sean Elliott, In-house Clinical Research Associate, Fortrea-FSP, UK; ^c^Adrian Kilcoyne, Chief Medical Officer, Cellectis, New York, USA; ^d^John Bolodeoku, Visiting Senior Lecturer, Centre for Pharmaceutical Medicine Research, Institute of Pharmaceutical Science, King’s College London, UK; ^e^Assem el Baghdady, Senior Lecturer, Centre for Pharmaceutical Medicine Research, Institute of Pharmaceutical Science, King’s College London, UK

**Corresponding author**

*Meena Arora

meenakarora@icloud.com

**Table S1. Eligibility criteria for the systematic search of ClinicalTrials.gov**

| PICOS | Inclusion Criteria | Exclusion Criteria |
| --- | --- | --- |
| Population | - All patients regardless of age, sex, ethnicity diagnosed with cancer (any type) | - Non-cancer studies |
| Interventions | - Any investigational drug or other product – medicine, medical device, vaccine or procedure | – |
| Comparators | - Any comparator, including standard of care practice, placebo or no comparator treatment | – |
| Outcomes | - Completed studies with/without results - Active studies, or those actively recruiting and those not currently recruiting | – |
| Study design | - Interventional studies (Phase 1 to Phase 4) randomised or non-randomised | - Systematic review and meta-analysis - Observational studies - Case reports/case series (with N ≥ 10) - Animal, in vitro studies - Pharmacokinetic studies - Review articles - Notes, editorial, correspondence, or opinion - Letters |
| Language | English | Non-English |
| Time period | 2011–2020 | |

**Table S2. Eligibility criteria for the systematic search of published literature**

| **Inclusion criteria** |
| --- |
| 1. **Focus on disease of interest:** Only publications that focused on a specific type of cancer or the field of oncology as a whole were included (e.g. any articles that focused on a disease other than cancer were excluded). 2. **Focus on the implications of Brexit on clinical research:** Only publications that focused on the impact that Brexit has had on the conduct of clinical research in the UK were included (e.g. any articles that did not address how Brexit has impacted clinical research in the UK were excluded). 3. **Geographical location:** Only publications that focused on the United Kingdom were included (i.e. any articles that did not relate to the UK were excluded). 4. **Format:** Only publications that had both an abstract and full text available to read were included (i.e. any articles with an abstract but no full text were excluded). 5. **Type of literature:** Only peer-reviewed research papers or reports from identified stakeholders were included (e.g. conference papers, websites, article reviews and personal opinion pieces were excluded). 6. **Duplicates:** Any copies of the same publication generated from the literature search were identified to ensure that no information was duplicated. |

**Table S3. Overview of CASP scores for included publications in the systematic literature review**

|  | **Studies included in systematic review** | | | | | | | | |
| --- | --- | --- | --- | --- | --- | --- | --- | --- | --- |
|  | **Appraisal criteria** | **SOIF (2018)** | **Tsagakis (2020)** | **Vousden (2018)** | **Matthews (2018)** | **Lawler (2018)** | **Breckenridge (2019)** | **Reijntjes (2018)** | **Hawkes (2018)** |
| **1** | Did the paper address a clearly focused question? | +1 | +1 | +1 | +1 | +1 | +1 | +1 | +1 |
| **2** | Did the authors search for the right type of papers? | +1 | +1 | +1 | +1 | +1 | -1 | +1 | +1 |
| **3** | Were all relevant papers included? | +1 | +1 | +1 | +1 | +1 | 0 | +1 | +1 |
| **4** | Did the authors sufficiently assess the quality of included papers? | +1 | +1 | -1 | +1 | -1 | 0 | +1 | +1 |
| **5** | If the results of the paper were combined, was this justified by the authors? | 0 | 0 | 0 | +1 | 0 | 0 | +1 | 0 |
| **6** | Are the overall results of the paper clear? | +1 | +1 | +1 | +1 | +1 | +1 | +1 | +1 |
| **7** | Are the overall results justified by the authors? | +1 | +1 | +1 | +1 | +1 | +1 | +1 | +1 |
| **8** | Can the results of the paper be applied to the population in question? | +1 | +1 | +1 | +1 | +1 | +1 | +1 | +1 |
| **9** | Were all important outcomes considered? | +1 | +1 | 0 | +1 | +1 | +1 | +1 | +1 |
| **10** | Is there a clear conclusion to the paper? | +1 | +1 | +1 | +1 | +1 | +1 | +1 | +1 |
| **Total score** | 9 | 9 | 6 | 10 |  | 8 | 5 | 10 | 9 |

SOIF, School of International Futures

|  | **Studies included in systematic review** | | | | |
| --- | --- | --- | --- | --- | --- |
|  | **Appraisal criteria** | **Mimoglu**  **(2016)** | **Selby**  **(2016)** | **Hatswell (2017)** | **Hervey**  **(2021)** |
| **1** | Did the paper address a clearly focused question? | +1 | +1 | +1 | +1 |
| **2** | Did the authors search for the right type of papers? | +1 | +1 | +1 | +1 |
| **3** | Were all relevant papers included? | -1 | -1 | 0 | +1 |
| **4** | Did the authors sufficiently assess the quality of included papers? | +1 | +1 | +1 | +1 |
| **5** | If the results of the paper were combined, was this justified by the authors? | 0 | 0 | 0 | 0 |
| **6** | Are the overall results of the paper clear? | +1 | +1 | +1 | +1 |
| **7** | Are the overall results justified by the authors? | +1 | +1 | +1 | +1 |
| **8** | Can the results of the paper be applied to the population in question? | +1 | +1 | +1 | +1 |
| **9** | Were all important outcomes considered? | +1 | +1 | +1 | +1 |
| **10** | Is there a clear conclusion to the paper? | +1 | +1 | +1 | +1 |
| **Total score** | 9 | 7 | 7 | 8 | 9 |

Key: 1=yes; -1=no, 0=not assessable

**Survey questionnaire**

| 1. | Please select which of the following organisations you work for. | ☐ MHRA  ☐ Multinational jnnpharmaceutical jnncompany  ☐ Contract Research jnnOrganisation (CRO)  ☐ Cancer charity  ☐ Freelance oncology jnn jnnconsultant  ☐ Academic institute jnn(e.g. university)  ☐ Other (please specify) |
| --- | --- | --- |
| 2. | Have you noticed a change in the annual number of oncology clinical trials conducted in the UK since the vote to leave the European Union (EU) in 2016? | ☐ Significant increase jnn(>25% more)  ☐ Slight increase jnn(10-25% more)  ☐ No noticeable change  ☐ Slight decrease jnn(10-25% less)  ☐ Significant decrease jnn(>25% less) |
| 3. | How confident are you that the UK will remain a world leader in oncology research outside of the EU? | ☐ Very confident  ☐ Fairly confident  ☐ No opinion  ☐ Fairly uncertain  ☐ Very uncertain |
| 4. | In your opinion, how will oncology research in the UK fare outside of the EU as opposed to the UK’s previous membership of the EU? | ☐ Significantly better  ☐ Slightly better  ☐ No noticeable change  ☐ Slightly worse  ☐ Significantly worse |
| 5. | How confident are you that cancer patients will continue to benefit from early access to investigational treatments, through participation in oncology clinical trials now that the UK has formally left the EU? | ☐ Very confident  ☐ Fairly confident  ☐ No opinion  ☐ Fairly uncertain  ☐ Very uncertain |
| 6. | Will there be a noticeable change in the degree of collaboration between the UK and other EU countries in oncology research following the UK’s withdrawal from the EU? | ☐ Significant increase  ☐ Slight increase  ☐ No noticeable change  ☐ Slight decrease  ☐ Significant decrease |
| 7. | In your opinion, how important is it for the UK to closely align itself with the upcoming Clinical Trials Regulation despite the UK no longer being a member of the EU? | ☐ Very important  ☐ Fairly important  ☐ No opinion  ☐ Fairly unimportant  ☐ Not important at all |
| 8. | Please select which of the following non-EU countries you would like the UK to seek closer collaborative ties with, in the field of oncology research? | ☐ USA  ☐ China  ☐ Japan  ☐ Canada  ☐ Australia  ☐ Brazil  ☐ South Africa  ☐ Other (please specify) |
| 9. | How confident are you that the potential loss of EU funding for future UK oncology research will be fully matched by the UK government? | ☐ Very confident  ☐ Fairly confident  ☐ No opinion  ☐ Fairly uncertain  ☐ Very uncertain |
| 10. | At present, how satisfied are you that the regulations for clinical trials that have been put in place in the UK, since leaving the EU, are sufficient to enable the UK to remain a world leader in oncology research? | ☐ Very satisfied  ☐ Fairly satisfied  ☐ No opinion  ☐ Fairly disatissfied  ☐ Very dissatisfied |

**Stakeholder mapping**

**MHRA:**

The MHRA is the most important stakeholder in the context of this research project as it has the greatest ability to enact policy changes in the future to help the UK remain a world leader in the field of oncology research. Moreover, considering that this project will be one of the most detailed and current pieces of work on the impact of Brexit on oncology research, it may help contribute to beneficial legislative and other changes for future oncology research.

The MHRA, as a now completely independent regulatory authority can also decide how closely the UK will adhere to Clinical Trials Regulation from the EU and will play a crucial role in determining how easy it will be for the UK to co-ordinate oncology trials with other EU countries in the years after Brexit.

**Cancer Trial Sponsors:**

It is important to receive the opinions from one/several of the pharmaceutical companies and Clinical Research Organisations (CROs) that are currently living with the ramifications of Brexit, as well as gauging their opinions about the prospects of oncology research in the future.

**Cancer Research UK:**

Cancer charities, such as Cancer Research UK, are similarly important, not least because they have perhaps the closest relationship with patients whilst they undergo cancer treatment. Cancer Research UK has been involved in ~5000 cancer clinical trials since 2006, ~70% of which involved collaboration between the UK and one or more other EU countries meaning they will have insight into the impact of Brexit on oncology research.

**Academic Organisations:**

Universities are involved in the conduct of many oncology trials in the UK and as with pharmaceutical companies, have a vested interest in ensuring that the UK remains a world leader in the field of cancer research. Perhaps unlike industry though, the academic sector is slightly less driven by the need to create commercially viable cancer treatments and as such, has a slightly lower level of influence than their commercial counterparts.

**Patient Advocacy Groups:**

As clinical trial endpoints become more patient-centric the role of patient advocacy groups in clinical trials is expanding. It is beneficial to hear from patient advocacy groups as they represent the interests of cancer patients and will be keen observers of the impact of Brexit on the number of clinical trials taking place in the UK and the access patients have to innovative therapeutics.

**Figure S1.** Stakeholder mapping in terms of relative levels of influence and interest.


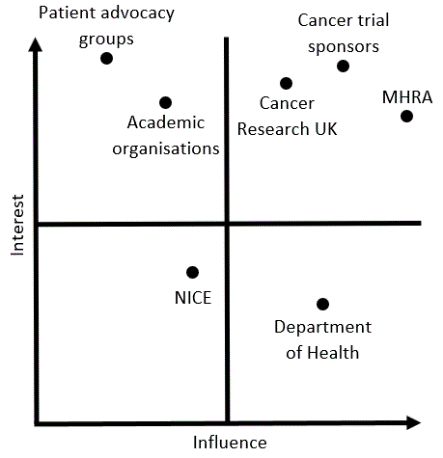


**REFERENCES**

Breckenridge A, Feldschreiber P. (2019). Impact of Brexit on UK and EU Drug Regulation and Patient Access. *Clin Pharmacol Ther,* 105(4):923-25. <https://doi:10.1002/cpt.1261>.

Hatswell AJ. (2017). How do we avoid disaster when exiting the European Medicines Agency? Making the most of Brexit in pharmaceutical regulation. *Ecancermedicalscience,* 11:ed67. <https://doi:10.3332/ecancer.2017.ed67>.

Hawkes N, Wise J, Armstrong S, et al. (2018). Braced for Brexit. *BMJ,* 363:k4724. <https://doi:10.1136/bmj.k4724>.

Hervey T, Antova I, Flear ML, et al. (2021). Health "Brexternalities": The Brexit Effect on Health and Health Care outside the United Kingdom. *J Health Polit Policy Law,* 46(1):177-203. <https://doi:10.1215/03616878-8706663>.

Lawler M, Begum M, Lewison G, et al. (2018). The impact of Brexit on UK cancer research. *Lancet Oncol,* 19(10):1276-78. <https://doi:10.1016/s1470-2045(18)30518–7>.

Matthews G, Williams N. (2018). Post-Brexit crystal ball gazing: The future for phase 1 clinical trials in the UK. *Med Leg J,* 86(2):63–71. <https://doi:10.1177/0025817218767265>.

Mimoglu ED, Kirca O, Ozdogan M. (2017). Cancer funding and Brexit. *J Oncological Sci,* 3:3-4. doi: <https://doi.org/10.1016/j.jons.2016.09.002>.

Reijntjes S, Albayaty M, Bush J, et al. (2018) The Association for Human Pharmacology in the Pharmaceutical Industry London Meeting 2018: Brexit and Other Challenges in Early Phase Drug Development. *Front Pharmacol,* 9:1301. <https://doi:10.3389/fphar.2018.01301>.

School of International Futures (SOIF) Cancer Research UK. (2021). Future of clinical trials after Brexit 2021 <https://www.cancerresearchuk.org/sites/default/files/future_of_clinical_trials_after_brexit.pdf>.

Selby P, Lawler M, Banks I, et al. (2016). The EU: what's best for UK cancer research and patients? *Lancet Oncol,* 17(5):556-7. doi: 10.1016/s1470-2045(16)30063-8.

Tsagakis I, Papatriantafyllou M. (2020). Safeguarding cancer research funding by European charities amidst the COVID-19 pandemic. *Mol Oncol,* 14(12):2987–93. <https://doi:10.1002/1878-0261.12839>.

Vousden KH. (2019). Brexit negotiations: what is next for science? *EMBO Rep,* 20(4) <https://doi:10.15252/embr.201948026>.
